# NanoIEA: A Nanopatterned Interdigitated Electrode Array‐Based Impedance Assay for Real‐Time Measurement of Aligned Endothelial Cell Barrier Functions

**DOI:** 10.1002/adhm.202301124

**Published:** 2023-11-02

**Authors:** Jong Seob Choi, Hyun Myung Doo, Byunggik Kim, Su Han Lee, Sang‐keun Sung, Gwangjun Go, Allister Suarez, Yeseul Kim, Byung Mook Weon, Byung‐Ok Choi, Hyung Jin Kim, Deok‐Ho Kim

**Affiliations:** ^1^ Department of Biomedical Engineering, Center for Microphysiological Systems Johns Hopkins University Baltimore MD 21205 USA; ^2^ Division of Advanced Materials Engineering Kongju National University Cheonan Chungnam 31080 South Korea; ^3^ Department of Health Sciences and Technology SAIHST Sungkyunkwan University Seoul 06351 South Korea; ^4^ Department of Biomedical Research Center Korea University Guro Hospital Seoul 08308 South Korea; ^5^ Division of Medical Oncology, Department of Internal Medicine Korea University Guro Hospital, Korea University College of Medicine Seoul 08308 South Korea; ^6^ Department of Mechanical Engineering Johns Hopkins University Baltimore MD 21218 USA; ^7^ Digital Health Care Research Center Gumi Electronics and Information Technology Research Institute (GERI) Gumi Gyeongbuk 39253 South Korea; ^8^ Department of Mechanical Engineering Chosun University Gwangju 61452 South Korea; ^9^ SKKU Advanced Institute of Nanotechnology (SAINT) School of Advanced Materials Science and Engineering Sungkyunkwan University Suwon 16419 South Korea; ^10^ Department of Neurology Samsung Medical Center Sungkyunkwan University School of Medicine Seoul 06351 South Korea; ^11^ School of Electrical and Electronic Engineering Ulsan College Ulsan 44610 South Korea; ^12^ Department of Medicine Johns Hopkins University School of Medicine Baltimore MD 21205 USA; ^13^ Institute for Nanobiotechnology Johns Hopkins University Baltimore MD 21218 USA

**Keywords:** endothelial barrier function, impedance assays, interdigitated electrodes, nanopatterned Nafion, poly (L‐DOPA)

## Abstract

A nanopatterned interdigitated electrode array (nanoIEA)‐based impedance assay is developed for quantitative real‐time measurement of aligned endothelial cell (EC) barrier functions in vitro. A bioinspired poly(3,4‐dihydroxy‐L‐phenylalanine) (poly (l‐DOPA)) coating is applied to improve the human brain EC adhesion onto the Nafion nanopatterned surfaces. It is found that a poly (l‐DOPA)‐coated Nafion grooved nanopattern makes the human brain ECs orient along the nanopattern direction. Aligned human brain ECs on Nafion nanopatterns exhibit increased expression of genes encoding tight and adherens junction proteins. Aligned human brain ECs also have enhanced impedance and resistance versus unaligned ones. Treatment with a glycogen synthase kinase‐3 inhibitor (GSK3i) further increases impedance and resistance, suggesting synergistic effects occur on the cell–cell tightness of in vitro human brain ECs via a combination of anisotropic matrix nanotopography and GSK3i treatment. It is found that this enhanced cell–cell tightness of the combined approach is accompanied by increased expression of claudin protein. These data demonstrate that the proposed nanoIEA assay integrated with poly (l‐DOPA)‐coated Nafion nanopatterns and interdigitated electrode arrays can make not only biomimetic aligned ECs, but also enable real‐time measurement of the enhanced barrier functions of aligned ECs via tighter cell–cell junctions.

## Graphical abstract

A nanopatterned interdigitated electrode array (nanoIEA)‐based impedance assay with a bioinspired poly (l‐DOPA) coating is developed for quantitative real‐time measurement of aligned endothelial cell barrier functions. GSK3 inhibition further increases impedance and resistance, suggesting synergistic effects occur on the cell–cell tightness of aligned endothelial cells via a combination of matrix anisotropy and GSK3i treatment.

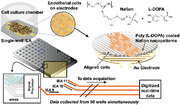

## 1 Introduction

For determination of cell–cell junctional barriers or integrity of in vitro endothelial cell (EC) layers, trans‐epithelial/endothelial electrical resistance (TEER) has been widely utilized in transwell platforms or electric cell–substrate impedance sensing with randomly seeded cell monolayers to study trans‐ or para‐cellular permeability.^[^ ^1^, ^2^, ^3^, ^4^, ^5^, ^6^, ^7^ ^]^ However, conventional in vitro culture systems do not recapitulate highly aligned endothelial structures and physiologically‐relevant barrier functions within in vivo models.^[^ ^8^ ^]^ It is well known that EC alignment mediated by fluid flow is recognized to be important for vascular barrier functions.^[^ ^9^, ^10^, ^11^ ^]^ In areas where the blood flow is laminar, vascular ECs are elongated and oriented longitudinally along the direction of flow, whereas the cells are polygonal and unaligned in disturbed flow regions.^[^ ^12^, ^13^ ^]^ Endothelial barrier dysfunction is substantially linked with cell morphological changes and the expression of adhesive proteins in the inter‐endothelial junctions.^[^ ^14^, ^15^ ^]^ Thus, previous studies have attempted to mimic aligned endothelial structures under flow dynamics or shear stress.^[^ ^16^, ^17^, ^18^, ^19^ ^]^ However, these approaches with flow dynamics or shear stress have limited applications to multiwell plates, microfluidics, or conventional cultureware since they require laminar, pulsatile, or oscillating flow‐controlled peristaltic devices.^[^ ^1^, ^20^, ^21^, ^22^, ^23^, ^24^, ^25^ ^]^ Thus, these approaches lack high‐throughput capability. Another approach is to use matrix topographical cues to align the cells where they are placed. For example, nanopatterned topographical surfaces have been fabricated to recapitulate nanofibrous collagen architectures in various in vitro applications to align the cells.^[^ ^26^, ^27^, ^28^, ^29^, ^30^, ^31^, ^32^ ^]^ It is well documented that nanotopographical matrix cues guide various cellular functions such as growth, proliferation, and differentiation compared to a conventional flat surface control condition.^[^ ^33^, ^34^, ^35^, ^36^ ^]^

Most nanotopographical surfaces for cell biological applications have been fabricated with insulating materials, such as polydimethylsiloxane (PDMS), polyurethane acrylate (PUA), or poly (lactic‐*co*‐glycolic acid) (PLGA).^[^ ^30^, ^37^, ^38^, ^39^, ^40^ ^]^ However, these materials block electrical current between cells and electrodes, which prevents measuring these signals. Our recent studies reported using Nafion, an ion‐permeable/conductive polymer, for the fabrication of nanopatterns and integration into electrodes for recording electrophysiology in human stem cell‐derived neurons and cardiomyocytes.^[^ ^41^, ^42^ ^]^ Unlike insulating materials, Nafion allows the electrical currents or potentials generated from living cells to move through the nanopatterned layer, facilitating real‐time measurement of electrophysiological signals from excitable cells.

Here we report a nanopatterned interdigitated electrode array (nanoIEA)‐based impedance assay for high‐throughput real‐time measurement of aligned endothelial cell barrier functions. We first fabricated Nafion grooved nanopatterns to align human brain ECs for mimicking cellular architectures in native endothelium in vitro.^[^ ^41^ ^]^ The Nafion polymer with nanogrooves was then integrated with an interdigitated electrode array (IEA) to quantitatively evaluate the barrier integrity of cell–cell junctions. The polymer of 3,4‐dihydroxy‐l‐phenylalanine (l‐DOPA), a dopamine precursor, was employed to modify the Nafion surface, thus enhancing the cell–substrate adhesion. The aligned EC layer shows higher expression of genes encoding tight and adherens junction proteins than is found within the unpatterned control group. Furthermore, we investigated the effects of a Glycogen synthase kinase‐3 inhibitor (GSK3i) on the cell–cell tightness of ECs by cell–substrate impedance sensing on the nanopatterned IEAs proposed in this study. The tightness of cell–cell junctional barriers modulated by GSK3i and measured by nanopatterned IEAs showed a good correlation with immunocytochemistry and western blot analysis. Incorporation of anisotropic nanopatterns of ion‐permeable Nafion into electrode array systems can provide cell culture environments as well as be appropriate for measuring real‐time cell–cell junctional barriers or integrity of ECs.

## 2 Results and Discussion

### 2.1 Fabrication and Characterization of Poly (l‐DOPA)‐Functionalized NanoIEA

**Figure 1:**
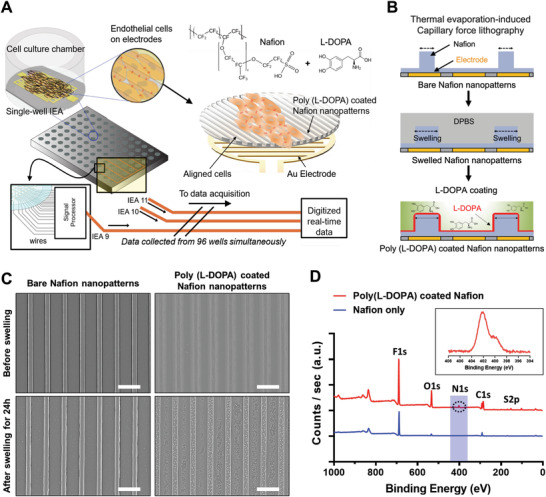
Design and characterization of IEA with poly (l‐DOPA) coated Nafion nanopatterns. A) Schematic illustration of high‐throughput nanoIEA concept. B) Fabrication steps to generate poly (l‐DOPA) coated nanopatterned Nafion films. C) SEM images of Nafion nanopatterns and poly (l‐DOPA) coated Nafion nanopatterns. D) XPS survey scan analysis for poly (l‐DOPA) coated Nafion films. Inset shows the magnification of N1s peak of poly (l‐DOPA) coated Nafion films. Scale bars: 2 µm.

Only a few researchers have adopted artificially synthesized Nafion as a biocompatible material because Nafion has been mostly developed and utilized for polymer electrolyte membrane fuel cells.^[^ ^43^ ^]^ These synthetic polymers usually need additional surface functionalization or modification with biocompatible materials such as collagen, fibronectin, or laminin before cell plating to make the cells better adhere to their surface. Since L‐DOPA is one of the dopamine derivatives and the key compound in the formation of marine adhesive proteins found in mussels, we introduced L‐DOPA as a cell adhesion promoter on the Nafion nanopatterns. **Figure** **1**A illustrates the design of the multiwell, nanotopographically patterned IEA devices, namely nanoIEA. We utilized commercially available 96‐well IEA plates. Each well of the 96‐well plate supports independent cell cultures. Within each well, the electrode recording system facilitates simultaneous real‐time recording of impedance generated by the aligned cells, then further analyzed through built‐in software. The fabrication steps are illustrated in Figure 1B. Briefly, Nafion nanopattern was fabricated using thermal evaporation‐induced capillary force lithography. Then, the Nafion nanopatterned layer was immersed and incubated in poly (l‐DOPA) solution. After overnight incubation at 37 °C, L‐DOPA became dark and thickened slightly. This may be attributed to the synthesis of L‐DOPA molecules into poly (l‐DOPA) nanoparticles. This can be seen in the SEM image analysis (Figure 1C). After aspirating L‐DOPA solution from the Nafion nanopatterned surface, a stacked and solidified poly (l‐DOPA) layer covered all the Nafion nanopatterns even though clear and narrow anisotropic nanopatterns were observed (Figure 1C, top panel). Figure S1, Supporting Information, shows the depth profiles of Nafion nanopatterns before/after poly (l‐DOPA) solution. Bare nanopatterns were ≈271 nm in height but decreased to ≈188 nm once poly (l‐DOPA) coated the Nafion nanopatterns. After washing the poly (l‐DOPA) layer, the height of nanopatterns returned to ≈271 nm. Interestingly, the morphologies of these Nafion nanopatterns were changed after swelling in dulbecco's phosphate‐buffered saline (DPBS) (Figure 1C, left, top and bottom). Bare Nafion nanopatterns were slightly swelled in the DPBS, resulting in wider ridges compared to bare nanopatterns. The ridge size changed from 401 ± 11 nm to 431 ± 19 nm. In addition, solidified poly (l‐DOPA) layers were rinsed out during the swelling and washing steps. In Figure 1C (right and bottom), synthesized poly (l‐DOPA) nanoparticles attached to nanopatterns on the Nafion surface. This indicates that L‐DOPA molecules can polymerize into nanoparticles and adhere to the Nafion surface. Furthermore, poly (l‐DOPA)‐coated Nafion nanopatterns showed a further increase in the width of the ridges. The ridge size was 472 ± 32 nm. Similarly, Nafion nanopatterns can swell by absorbing ions and water molecules into the Nafion molecule chains. Thus, Nafion nanopatterns were thickened by coating with poly (l‐DOPA) layer. Additionally, successful deposition of poly (l‐DOPA) on Nafion substrate was confirmed using an X‐ray photo electron spectroscopy (XPS)survey scan analysis (Figure 1D). A small peak around 400 eV, which corresponds to N1s on Nafion nanopattern films was present while bare Nafion showed no peaks near 400 eV. Poly (l‐DOPA) has Nitrogen atoms on its molecular backbones.

### 2.2 Effect of Poly (l‐DOPA) on Adhesion of hCMECs on the Nafion Nanopatterns

**Figure 2:**
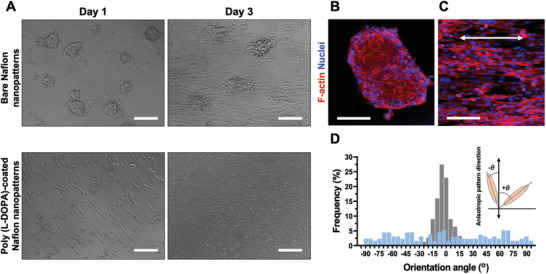
Effects of poly (l‐DOPA) on hCMEC adhesion onto Nafion nanopatterns. A) Bare Nafion nanopatterns at day 1 (top, left) and day 3 (top, right), poly (l‐DOPA) coated Nafion nanopatterns at day 1 (bottom, left) and day 3 (bottom, right). Confocal microscope images of B) an hCMEC aggregate on bare Nafion nanopatterns, and C) patterned hCMECs on poly (l‐DOPA) coated Nafion nanopatterns. D) Quantitative analysis of orientation angle of aligned (gray bar) and randomly oriented (sky‐blue bar) hCMECs on the Nafion layer. Scale bars: 100 µm.

**Figure** **2** shows the effects of poly (l‐DOPA) on the adhesion of human cerebral microvascular endothelial cells (hCMECs) onto the Nafion nanopatterns. The hCMECs tend to prominently aggregate at day 1 after plating as shown in Figure 2A (top and left). These aggregates make the hCMECs layer uneven after confluency (Figure 2A, top and right). This phenomenon can be explained by relatively stronger cell–cell adhesion compared with cell–substrate (Nafion) adhesion because Nafion is a synthetic polymer and nanostructures render hydrophobic properties (Figure S2, Supporting Information) during the initial stage of cell adhesion. However, this effect can be alleviated by poly (l‐DOPA) deposition onto the Nafion layer. Single hCMECs are uniformly spread and attached to poly (l‐DOPA) coated Nafion nanopatterns (Figure 2A, bottom and left). Poly (l‐DOPA) on the Nafion layer promotes strong cell–substrate interaction, allowing a well‐aligned and oriented hCMECs layer to be formed after confluency (Figure 2A, bottom and right). Figure 2B,C and Figure S3, Supporting Information, show confocal microscopy images of endothelial cells for better visualization. Aggregates on the bare Nafion layer (Figure 2B) were observed. However, well‐attached but randomly aligned hCMECs on the poly (l‐DOPA) coated unpatterned Nafion layer (Figure S3, Supporting Information), and well‐attached and aligned endothelial cells on the poly (l‐DOPA) coated nanopatterned Nafion layer (Figure 2C) were obtained by staining with dyes, respectively. Further, the orientation angle of hCMECs was calculated by the long axis of cells toward the anisotropic nanopattern direction (Figure 2D). More than 96% and 98% of hCMECs are aligned within 20° and 25°, respectively.

### 2.3 Enhanced Tight and Adherens Junction Gene Expression in hCMECs on Nanopatterned Nafion Layer

**Figure 3:**
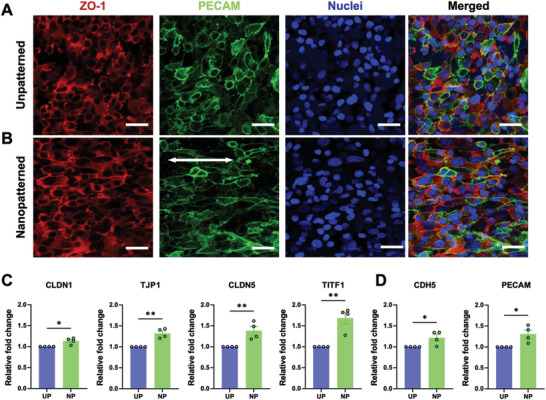
Differences in expression of cell–cell junction markers in hCMECs on Nafion layer. Immunocytochemistry of hCMECs on A) unpatterned (UP) Nafion, and B) nanopatterned (NP) Nafion. Junctional proteins (ZO‐1 and PECAM) and nuclei were stained, respectively. The white arrow indicates the direction of anisotropic Nafion nanopatterns. qRT‐PCR analysis of changes in expression of genes encoding C) tight and D) adherens junction proteins. *p < 0.05, **p < 0.01. Scale bars: 30 µm.

Brain microvascular ECs form a semi‐permeable barrier to control the transport of blood, solutes, and cells across vessel walls in the brain. The brain barrier is maintained by cell–cell integrity, including tight junctions, adherens junctions, and various other adhesion molecules.^[^ ^44^, ^45^ ^]^ The tight and adherens junctions influence the paracellular tightness of the endothelial cells and the permeability across vascular endothelial barrier function.^[^ ^24^ ^]^ Zonula occludens‐1 (ZO‐1) and platelet endothelial cell adhesion molecule (PECAM) are two of the dominant junctional proteins in ECs, regulating other junctional components, cell–cell tension, angiogenesis, endothelial barrier function, and formation.^[^ ^46^, ^47^ ^]^ In addition, multiple studies introduced anisotropically straightened nanopatterns in vitro to recapitulate in vivo‐like cellular environments.^[^ ^26^, ^28^, ^29^, ^30^ ^]^ **Figure** **3** shows differences in the gene expression in hCMECs on nanopatterned Nafion layers. Junction proteins such as ZO‐1 and PECAM were immunostained to observe the protein expression. However, the significant expression level via confocal microscopy of ZO‐1 and PECAM was not observed on either unpatterned or nanopatterned Nafion layers (Figure 3A,B). We only observed cellular alignments along the nanopattern direction (white arrow in Figure 3B). We also attempted to acquire 3D Z‐stack images of tight junction proteins. However, we encountered challenges due to interference caused by the Nafion materials and the relatively poorer vertical resolution of the confocal microscope compared to its lateral resolution. Consequently, we were unable to obtain representative images using this approach. Considering the aforementioned technical difficulties, we did not include imaging‐based quantification of protein expression between NP and UP samples.

On the other hand, the qRT‐PCR analysis showed distinguishable differences in gene expression encoding tight and adherens junction proteins. We found that the genes related to tight junction proteins such as CLDN1, TJP1, CLDN5, and TITF1 were significantly expressed in hCMECs on the Nafion nanopatterns (Figure 3C). Further, the genes encoding adherens junction proteins such as CDH5 and PECAM were also significantly expressed in this condition. In summary, Nafion nanopatterns contributed to enhancing gene expression for tight and adherens junction proteins.

### 2.4 Electrochemical Impedance Analysis and Current Flow Simulation of Nanopatterned Nafion Electrodes

**Figure 4:**
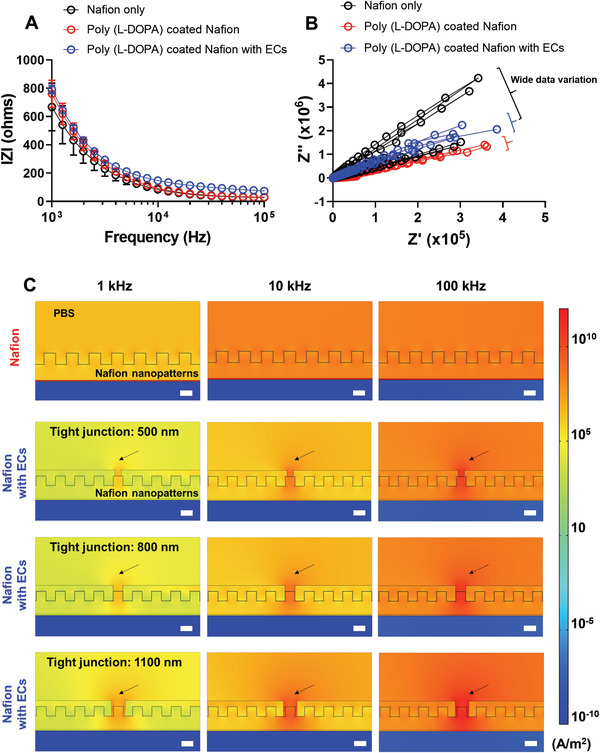
Electrochemical impedance spectroscopy and electrical current flow simulation study of nanopatterned Nafion electrodes. A) Total impedance behaviors versus frequency. B) The Nyquist plots (imaginary *Z* versus real *Z*). C) Electrical current distribution behaviors near cell–cell junctions in the different frequencies (1–100 kHz) and tight junction distances (500, 800, and 1100 nm). Black arrows indicate concentrated electrical current density near the cell–cell junction. Scale bars: 1 µm.

Impedance‐based cell–cell junction monitoring requires robust frequency response. We adopted electrochemical impedance spectroscopy (EIS) to investigate the frequency‐dependent electrochemical characteristics of poly (l‐DOPA) coated Nafion layer (**Figure** **4**). At the lower frequencies (Figure 4A), the Nafion‐only electrode layer showed an unstable impedance response represented by the wide error bars when compared to the poly (l‐DOPA) coated Nafion electrode. This indicates that poly (l‐DOPA) stabilizes the electrical current path over the Nafion layer at a lower frequency. At higher frequency spectrum where the electrical interface starts to behave like a conductor, the impedance response also stabilized. This trend was further confirmed by the Nyquist plot (imaginary Z versus real Z) (Figure 4B). Nafion‐only layers showed wide data variation rather than the poly (l‐DOPA) coated Nafion electrode and the electrical conductivity, represented by the slope, is lower. In addition, seeding hCMECs on nanoIEA did not affect the impedance response of nanoIEA. We further confirmed this impedance behavior by simplified computational modeling, using COMSOL, of the electrical current flow and charge density mapping near cell–cell boundaries on the nanopatterned Nafion electrodes. We fixed multiple layers consisting of a glass substrate, Au electrode pairs, nanopatterned Nafion layer, electrical double layers, cells, and phosphate‐buffered saline (PBS) (Figure S4, Supporting Information). The distance of cell–cell junctions was fixed to 500, 800, and 1100 nm in this study.^[^ ^48^ ^]^ Electrical current flow and density map are consistently presented when no cells are layered on the Nafion electrode (Figure 4C, Top panel). However, when the cells and cell–cell junctional gaps exist on the nanopatterned Nafion electrodes, the electrical density distribution map near the cell–cell gap junctions can be changed depending on the distance of the cell–cell junction and input electrical frequencies. The wide space of junctions between cells makes the electrical current density more concentrated near the cell–cell gap junctions. The electrical current density near the cell–cell gap junctions can be changeable and distributed to the PBS region with changing frequency ranges. In the low frequencies of 1 kHz, our computational model showed trapped electrical currents near the cell junctions, resulting in unstable electrical movement and inaccurate impedance and resistance values. However, this phenomenon can be alleviated by a higher frequency range, as depicted in the figure for 10–100 kHz.

### 2.5 Real‐Time Impedance Analysis of Cell–Cell Junction Integrity in hCMEC Monolayers with GSK3i on NanoIEA

**Figure 5:**
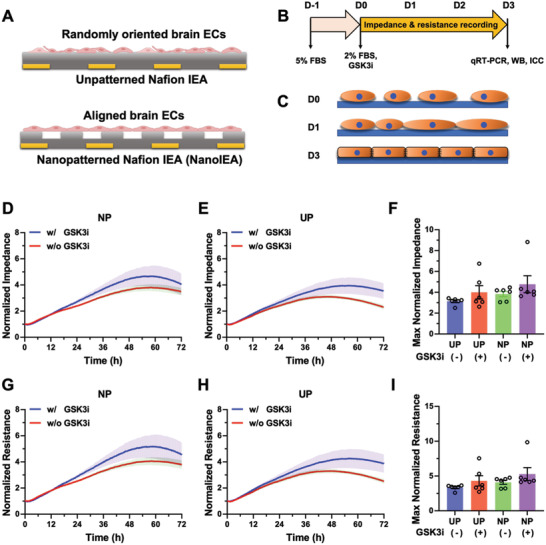
Quantitative measurement of cell–cell junction integrity in hCMEC monolayers on nanoIEA under GSK3 inhibitor treatment. A) Illustration of unpatterned (UP) Nafion IEA and nanopatterned (NP) Nafion IEA (nanoIEA). B) Timeline of impedance or resistance recordings with nanoIEA. C) Schematics of cell–cell junction formation on nanoIEA. D–F) Real‐time monitoring of impedance changes at high frequency (41 kHz). G–I) Real‐time monitoring of resistance changes. F,I) Max normalized impedance and resistance.

There has been no perfect in vitro brain EC physical barrier model reproducing the in vivo environment. Most traditional in vitro EC physical barrier models do not have sufficient tightness compared to in vivo EC barrier. So, it is important to carefully choose the in vitro EC physical barrier model and measurement system according to the requirements of the study and interpretation of the data.^[^ ^49^ ^]^ In previous literature, Marlyn et al. reported improved in vitro hCMEC physical barrier by treatment with LiCl, a GSKi that prevents activation of the β‐catenin destruction complex, resulting in reduced paracellular permeability. This result indicates that treatment with GSKi tightened the EC physical barrier.^[^ ^50^ ^]^ Here, our proposed nanoIEA was evaluated by recording real‐time impedance or resistance signals to investigate how GSK3i affects in vitro hCMEC physical barrier (**Figure** **5**). The ion‐permeable Nafion nanopattern layer was integrated with an interdigitated electrode array to record the tightness of cell–cell junctional barriers or integrity as they mature (Figure 5A). Nafion‐nanopatterned 96‐well interdigitated electrode plate (nanoIEA) was prepared for the high‐throughput screening. Optimized Nafion nanopatterns with poly (l‐DOPA) were layered on the commercialized interdigitated electrode (IEA) plate. Then, hCMECs were cultured on nanoIEA according to the experimental timeline (Figure 5B). On day −1, 50k endothelial cells were plated on the 96‐well nanoIEA plate. The concentration of FBS was reduced to 2%, and GSK3i was added at day 0 to prevent further proliferation and growth of hCMECs as well as induce matured cell–cell tightness of immortalized hCMECs. Single hCMECs were sparsely attached at day −1 (Figure 5C). Then, 80–90% confluency was achieved the next day (Day 0). hCMECs further grew and proliferated until day 2 to 3, where they formed maximum tightness. Quantitative impedance and resistance of hCMECs on nanopatterns slightly increased compared to hCMECs on unpatterned Nafion IEA (Figure 5D,E,G,H), which means aligned hCMECs on the nanopatterns have slightly higher cell–cell tightness compared to randomly cultured hCMECs. In addition, the effects of GSK3i on EC tightness also was confirmed with our proposed nanoIEA. The addition of GSK3i to the hCMECs causes higher impedance and resistance signals during the formation of tightness in the hCMECs layer. Nanopatterned physical cues and GSK3i showed synergistic effects on the cell–cell junctional barriers or integrity of in vitro hCMECs layers (Figure 5F,I).

We further investigated the effects of frequency ranges when measuring the cell–cell junctional barriers or integrity of in vitro hCMECs layers on the nanoIEA (Figures S5–S7, Supporting Information). All conditions were the same in Figure 5. However, different frequencies were tested to observe the effects on impedance and resistance measurements. Unstable and inconsistent impedance or resistance values during the recording were obtained at a lower frequency (1 kHz) (Figure S5, Supporting Information). Low‐frequency measurement seems to be interrupted by the formed cell layers while high‐frequency measurements showed that impedance and resistance are relatively stable, allowing for accurate cell barrier functions. Impedance and resistance change when the no cell (blank controls) is further investigated to the IEA (Figure S6, Supporting Information). Negative values over time were observed at low‐frequency measurements, which come from unstable electrical movement between the electrodes. However, this can be alleviated by enhancing input frequencies (Figure S6B,C,E,D, Supporting Information).

In addition, this impedance or resistance trend showed consistency and stability by enhancing frequency (10 kHz) as cells grow as shown in Figure S7, Supporting Information. These results are quite consistent with the computational modeling analysis in Figure 4C. The measurement sensitivity of nanoIEA can be enhanced by increasing recording frequencies.

**Figure 6:**
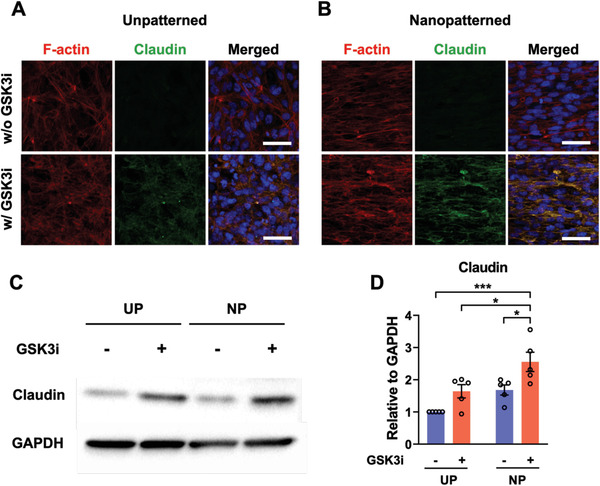
Effects of GSK3 inhibition on cell–cell junction protein expression in hCMECs on nanopatterned Nafion layer. A,B) Immunocytochemistry of F‐actin and claudin in hCMECs. C) Western blot for claudin. Quantitative analysis of protein expression of D) claudin in the hCMEC on unpatterned (UP) and nanopatterned (NP) Nafion layer. Scale bars: 50 µm.

We performed confocal microscopy image analysis and western blots to quantitatively analyze the effects of GSK3i on cell–cell junction protein expression in hCMECs (**Figure** **6**). Representative junctional proteins such as ZO‐1, VE‐cadherin, and claudin were immunostained and compared before/after GSK3i treatment. These proteins are rarely expressed without GSK3i treatment. Treating the ECs with GSK3i enhances the expression of these junctional proteins (Figure 6A,B; Figure S8, Supporting Information). In particular, Claudin, constituting the claudin family, shows remarkable expression after GSKi treatment. Claudin has been demonstrated to be the endothelial cell‐specific component of tight junction strands and is also predominantly expressed in BBB tight junctions.^[^ ^51^, ^52^ ^]^ The western blot analysis (Figure 6C,D) further supports this result. Synergistic effects of the nanopatterned layer and GSK3i were observed for claudin expression. Nanopatterns enhanced the expression level of claudin when compared to hCMECs on the unpatterned Nafion layer. The expression of this protein was further raised after GSK3i treatment on the hCMECs layer. This result suggests that GSK3i treatment may make cell–cell junctions of hCMECs more robust with synergistic effects with the Nafion nanopatterned layer.

**Figure 7:**
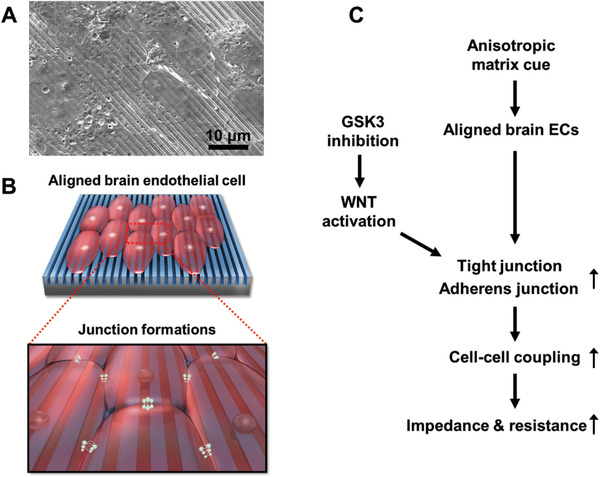
Model of brain EC signaling on nanopatterned Nafion. A) SEM image of aligned brain ECs on (l‐DOPA)‐coated Nafion nanopatterned substrate. B) Illustration of cell–cell junctions of aligned brain ECs grown on poly (l‐DOPA)‐coated Nafion nanopatterned substrate. Junctions produced in cell–cell contact areas are indicated in green. C) GSK3i and nanopatterned Nafion synergistically increase cell barrier function through proposed cell signaling pathways. Impedance and resistance measurements are evaluated using nanoIEA.

We have demonstrated in human brain ECs that enhanced barrier tightness can be achieved by the inactivation of GSK3. Our new IEA device integrated with nanopatterns is capable of real‐time impedance analysis to assess the stability of the cell barrier function. We also provide scanning electron microscope (SEM) images showing the cell's filopodia and lamellipodia aligning along the nanoscale grooves compared to the unpatterned surface. Endothelial cells are elongated and aligned along the nanogroove direction, as shown in Figure S9, Supporting Information. We could identify the endothelial cell's lamellipodia at the edge protruding into nanogrooves as depicted in Figure S9A, Supporting Information. In contrast, cells on a flat unpatterned surface showed randomly protruding lamellipodia and filopodia (Figure S9B,C, Supporting Information). In the hCMECs aligned on the nanopattern, the WNT signaling pathway is activated through GSK3i treatment, which increases the expression of tight junction and adherens junction related genes and proteins, resulting in greater cell–cell coupling. This cell–cell coupling enables real‐time quantitative measurement with the nanoIEA (**Figure** **7**, summary of mechanism).

## 3 Conclusion

A nanoIEA‐based impedance assay with a bioinspired poly (l‐DOPA) coating is developed for quantitative real‐time measurement of aligned endothelial cell barrier functions in vitro. We introduced poly (l‐DOPA)‐coated Nafion nanogroove patterns to orient the hCMECs along the nanogroove direction and promote strong cell adhesion onto Nafion nanopatterns. Aligned hCMECs on Nafion nanopatterns showed enhanced expression of genes encoding tight and adherens junction proteins. Poly (l‐DOPA)‐coated Nafion nanopatterns were further integrated with the IEA system for quantitative real‐time impedance assays of in vivo‐like aligned hCMECs barrier functions. The effects of GSK3i were simultaneously investigated for evaluating proposed nanoIEA devices. Aligned hCMECs showed enhanced impedance and resistance values. GSK3i treatment showed further increased impedance and resistance values, which means synergistic effects occur on the cell–cell tightness of in vitro hCMECs by using a combination of nanopatterns and GSK3i treatment. Our approach, combining nanopatterned substrate and GSK3i treatment, significantly increased cell–cell tightness of in vitro ECs by enhancing the expression of junctional proteins. The nanoIEA developed in this study provides a platform for ECs to form more mature cell–cell junctional barriers and evaluates the integrity of in vitro hCMECs layers.

## 4 Experimental Section

### Fabrication of Nanopatterned Nafion Layer

Nafion nanostructures were fabricated as described in previous literature.^[^ ^42^, ^53^ ^]^ First, nanopatterned PUA molds were prepared from a silicon (Si) master mold with anisotropic 800 nm (ridge and groove) and 600 nm (depth) dimensions. Si master mold substrate was custom‐made from National NanoFab Center (South Korea). The UV‐curable polymer precursor (MINS‐301RM, Minuta tech.) was dropped onto the Si mold and covered with a polyethylene terephthalate (PET) film. The UV‐curable polymer precursor was then homogeneously spread to avoid defects and make an even surface. Under 1 min. UV irradiation, the polymer precursor was crosslinked and then peeled from the Si master mold. Using this secondary cured polymer mold, the PDMS mold was prepared. PDMS mixtures with 10:1 weight ratio (PDMS prepolymer: curing agent) were poured onto secondary master mold, degassed, and cured in an oven. The 5% Nafion solution was dropped on precleaned glass cover substrate and pressed with a patterned PDMS mold. After drying for 3 days, the PDMS master mold was carefully removed from glass cover substrate.

### Electrochemical Impedance Spectroscopy Measurement

Impedance properties of nanopatterned Nafion layers were measured on an Interface 1010E (Gamry Instrument) equipped with an EIS module. The frequency employed for measurements ranged from 1 to 100 kHz with an AC amplitude of 1 mV. The interdigitated electrode array were fabricated via photolithography. A negative photoresist was spin‐coated and patterned under UV mask aligner to define the electrode patterns and then 20 nm of Cr and 200 nm of Au were layered in sequence by a thermal deposition. The negative photoresist was lifted off by gentle shaking in acetone. The final width and space between electrodes were 120 µm, respectively. To confine the Nafion and electrolyte solution on the interdigitated electrode, PDMS wells were fabricated to enclose each interdigitated electrode area. A PDMS sheet was prepared by mixing PDMS prepolymer and curing agent (10:1 weight ratio), degassed to remove bubbles, and placed in 65 °C oven to cure overnight. Each well was then created by using a 6 mm hole puncher on the cured PDMS sheet and attached to each interdigitated electrode. To pattern Nafion layer onto the electrodes, 5% Nafion solution was dropped inside PDMS well and pressed with a pre‐patterned PDMS mold. After drying for 3 days, the PDMS mold was removed from the surface of electrode, leaving a nanopatterned Nafion layer. The PDMS well was filled with DPBS to wet the patterned Nafion layer.

### Characterization of Poly (l‐DOPA)‐Coated Nanopatterned Nafion Layer

Nanopatterned Nafion substrates were coated with 3,4‐dihydroxy‐l‐phenylalanine (l‐DOPA; Sigma‐Aldrich).^[^ ^30^ ^]^ A 2 mg mL^−1^ working solution was generated by dissolving L‐DOPA in 10 mm Trizma Pre‐set crystals (Sigma‐Aldrich) with a pH of 8.5. Samples were then immersed and incubated at 37 °C overnight. Afterward, the L‐DOPA solution was aspirated, and substrates were washed with DPBS. Scanning electron microscopy (SEM; ZEOL, JSM‐7610F) was used for analyzing the topography of Nafion nanopatterns. Nanopattern depth/height was measured by atomic force microscope (AFM; HITACHI AFM5300E Scanning Probe Microscope) with a Si‐DF40 probe at a 0.38 Hz scan rate. XPS (ESCALAB 250, Thermo Scientific) equipped with a micro‐focused Al K‐α source was used for chemical analysis of L‐DOPA. The base pressure was ≈2–3 × 10^−8^ mbar.

### Cell Culture and Immunocytochemistry Analysis

The brain microvascular endothelial cell line hCMEC/D3 (SCC066, Merck) was purchased. hCMECs were cultured and maintained in EndoGRO‐MV Complete Media Kit (Cat. No. SCME004) supplemented with 5 ng mL^−1^ FGF‐2. For immunocytochemistry analysis, cells were fixed in 4% paraformaldehyde for 20 min at room temperature, then blocked and permeabilized in blocking buffer (5% Donkey serum, 0.1% FBS, 0.1% Triton X‐100 in phosphate buffered saline) for 15 min at room temperature. Cells were stained with Alexa Fluor 488 conjugated with CD144 (VE‐cadherin) monoclonal antibody (Thermo Fisher Scientific), Alex Fluor 594 conjugated with ZO‐1 monoclonal antibody (Invitrogen), Alexa Fluor 488 conjugated with claudin‐5 antibody (Invitrogen), Alexa Fluro 488 or 594 phalloidin (Invitrogen), and Alexa Fluor 488 conjugated with CD31 (PECAM) monoclonal antibody (Invitrogen) diluted in blocking buffer, then incubated for 1 hour at room temperature. Nuclei were stained with DAPI (BioLegend) diluted in blocking buffer for 5 min. Fluorescent images were acquired with 20× or 40× objectives under a Zeiss LSM 700 or 780 confocal microscope.

### Impedance and Resistance Analysis of Cultured Endothelial Cells

To incorporate the nanopattenred Nafion layer into a 96‐well plate with IEA (CytoView‐Z, Maestro Edge, Axion Biosystems, USA), a 2.5 µL of 5% Nafion solution was dropped inside each well of IEA plate and pressed with a patterned PDMS stamp 5 mm in diameter. After drying for 3 days in an air flow safety cabinet, the PDMS master mold was carefully removed from the wells of IEA plate. Each well was UV‐sterilized for 5 min, then 2 mg mL^−1^ of L‐DOPA solution was used for Nafion surface modification, and incubated at 37 °C overnight. Afterward, the L‐DOPA solution was aspirated and washed with DPBS. 50 000 cells were plated in each well. Impedance and resistance while culturing the cells were recorded by a commercially available software program (Axion Biosystems).

### qRT‐PCR Analysis

Total RNA was isolated using the RNeasy Mini Kit (Qiagen). The cDNA was synthesized using SuperScript III Reverse Transcriptase (ThermoFisher). A total of 1 µL template cDNA, 5 µL SYBR‑ Green PCR master mix (Applied Biosystems; Thermo Fisher Scientific, Inc.) and 10 pmol (total volume, 10 µL) of each primer was added to the qPCR reaction. The following thermocycling conditions were used for qPCR: 3 min at 95 °C, followed by two‑step PCR at 95 °C for 15 s and 60 °C for 60 s for 40 cycles, with fluorescence monitoring at the end of each elongation step. Gene expression was quantified using the 2^−ΔΔCt^ methods. GAPDH was used as the endogenous reference gene. Primer sequences are in Table S1, Supporting Information.

### Western Blot Analysis

Cells were washed in DPBS (Gibco) and then lysed on ice for 15 min in lysis buffer (M‐PER Mammalian Protein Extraction Reagent, ThermoFisher) with a 1× protease/phosphatase inhibitor cocktail (Cell Signaling Technology). Lysates were centrifuged for 20 min at 10 000 g, 4 °C and protein concentrations were estimated using the Pierce BCA Protein Assay Kit (ThermoFisher). Equal amounts of protein were electrophoresed on NuPAGE 4–12% Bis‐Tris Gels (Invitrogen) and transferred onto Polyvinylidene fluoride membranes (Bio‐rad). Membranes were probed with primary antibodies to Claudin‐1 (rabbit monoclonal, 1∶1000, Cell Signaling Technology), Occludin (rabbit monoclonal, 1∶1000, Cell Signaling Technology), and GADPH (rabbit, 1:5000, Cell Signaling Technology) overnight at 4 °C. Membranes were then incubated with anti‐rabbit secondary antibodies (1∶5000, Cell Signaling Technology) for 1 h at RT before visualization using a ChemiDoc imaging system (Bio‐rad). Integrated optical densities of the immunoreactive protein bands were measured using ImageJ software.

### Numerical Simulation of Electrical Current Flow Pathways

A 2D numerical simulation was performed via COMSOL Multiphysics Modelling Software to investigate the electric current flow pathways. In this model, the nanopatterned electrode with Nafion layer interface was designed to consist of a glass substrate, gold electrodes (thickness: 120 nm and space: 120 nm), nanopatterned film (thickness: 2 µm), and PBS. The dielectric properties of Au electrodes, the glass substrate, and PBS was determined from the material library of the COMSOL software. The electrical conductivity of Nafion nanopatterned film, cell, and cell membrane were set to 1.4 S m^−1^,^[^ ^54^ ^]^ 0.7 S m^−1^,^[^ ^55^ ^]^ and 1 × 10^−8^ S m^−1^,^[^ ^55^ ^]^ respectively. Intercellular junctions between two neighboring cells were set to be 800 nm.^[^ ^48^ ^]^ An electrical double layer (thickness: 2 nm and electrical conductivity: 2.710 × 10^−9^ S m^−1^) was applied to boundaries of gold electrodes and nanopatterned film contacted with PBS.^[^ ^56^ ^]^ The size of the nanopatterns was set as 800 nm ridges, 800 nm grooves, and 800 nm depth. An alternating voltage with a peak‐to‐peak of 1 V in the frequency range 1–100 kHz was applied to the electrode.

### Contact Angle Analysis

Contact angles were measured using a sessile drop method (KRUSS, DSA100L Drop Shape Analyzer). A water droplet (20 µL) was deposited on the nanopatterned surfaces and imaged with a camera to measure the contact angles. The contact angles were automatically measured by the ellipse (Tangent‐1) fitting method.

### Statistical Analysis

All quantitative data are presented as mean ± standard error of the mean (S.E.M.) (*n* ≥ 3, independent experiments). All statistical analyses were performed with GraphPad Prism 9. One‐way ANOVA with Tukey's posthoc multiple comparisons method was used to analyze data sets that included more than two experimental groups, while an unpaired two‐tailed *t*‐test was used to compare data sets looking at only two variables. In all analyses, a *p*‐value less than 0.05 was considered significant, and *n* was defined by the number of discrete cultured substrates.

## Conflict of Interest

D.‐H.K. is a scientific founder and equity holder of Curi Bio.

## Supporting information

Supporting Information

## Data Availability

The data that support the findings of this study are available from the corresponding author upon reasonable request.
