# Supplementary material for: NanoIEA: A Nanopatterned Interdigitated Electrode Array‐Based Impedance Assay for Real‐Time Measurement of Aligned Endothelial Cell Barrier Functions
Source: Adv Healthc Mater. 2023 Nov 2;13(2):2301124. doi: 10.1002/adhm.202301124 (PMC10841753; doi:10.1002/adhm.202301124)
Supplement: Supplementary file 1 — Supporting Information [file ADHM-13-2301124-s001.pdf]

ADVANCED HEALTHCARE MATERIALS

Supporting Information

for *Adv. Healthcare Mater.*, DOI 10.1002/adhm.202301124

NanoIEA: A Nanopatterned Interdigitated Electrode Array-Based Impedance Assay for Real-Time Measurement of Aligned Endothelial Cell Barrier Functions

*Jong Seob Choi, Hyun Myung Doo, Byunggik Kim, Su Han Lee, Sang-keun Sung, Gwangjun Go, Allister Suarez, Yeseul Kim, Byung Mook Weon, Byung-Ok Choi, Hyung Jin Kim and Deok-Ho Kim**

Supporting Information

NanoIEA: A Nanopatterned Interdigitated Electrode Array-based Impedance Assay for Real-time Measurement of Aligned Endothelial Cell Barrier Functions

Jong Seob Choi [†], *Hyun Myung Doo* [†], *Byunggik Kim, Su Han Lee, Sang-keun Sung, Gwangjun Go, Allister Suarez, Yeseul Kim, Byung Mook Weon, Byung-Ok Choi, Hyung Jin Kim, and Deok-Ho Kim*[*]

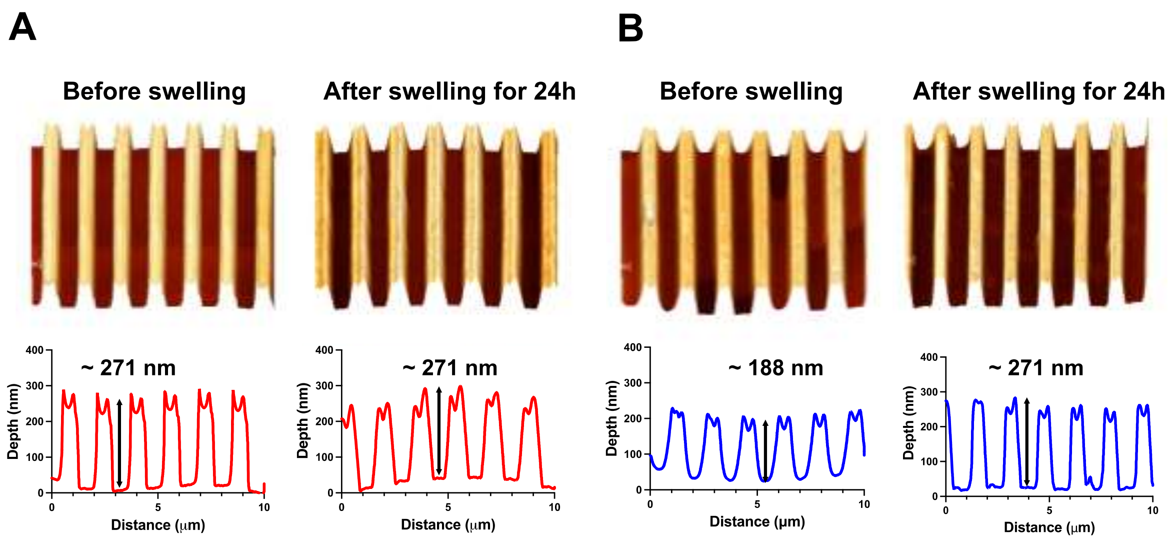

Figure S1. Depth profiles of (A) Nafion nanopatterns and (B) poly (L-DOPA)-coated Nafion nanopatterns.

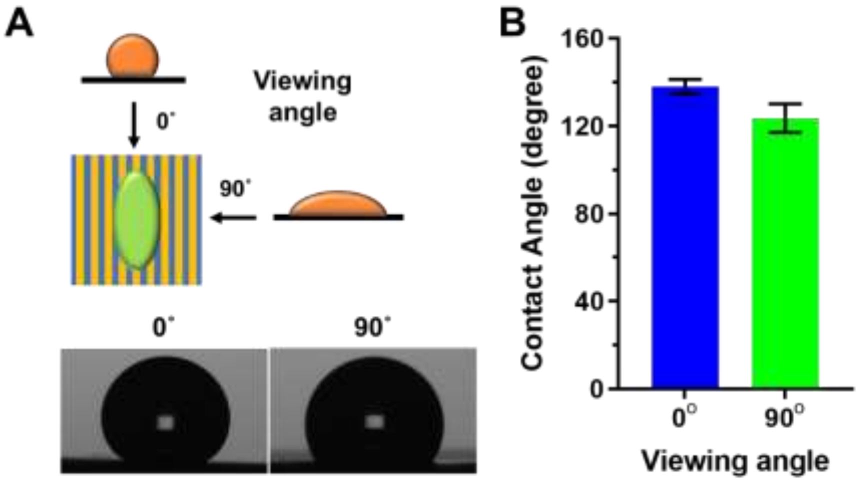

Figure S2. Water contact angles with regards to Nafion nanogroove direction. (A) Representative images of water droplets on nanopatterned Nafion films when viewed along and orthogonally to nanogrooves. (B) Measured contact angles of droplets on nanopatterned Nafion films.

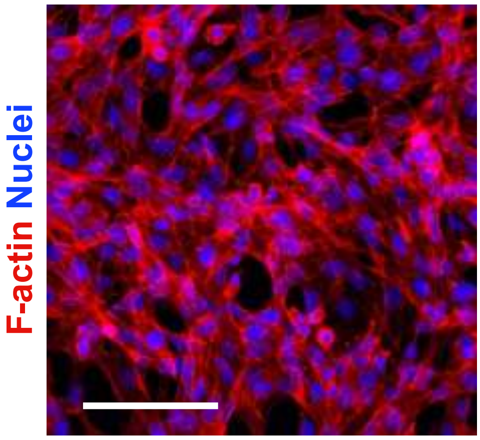

Figure S3. Randomly spread hCMECs on poly (L-DOPA) coated flat Nafion layer. Scale bar: 100 μm.

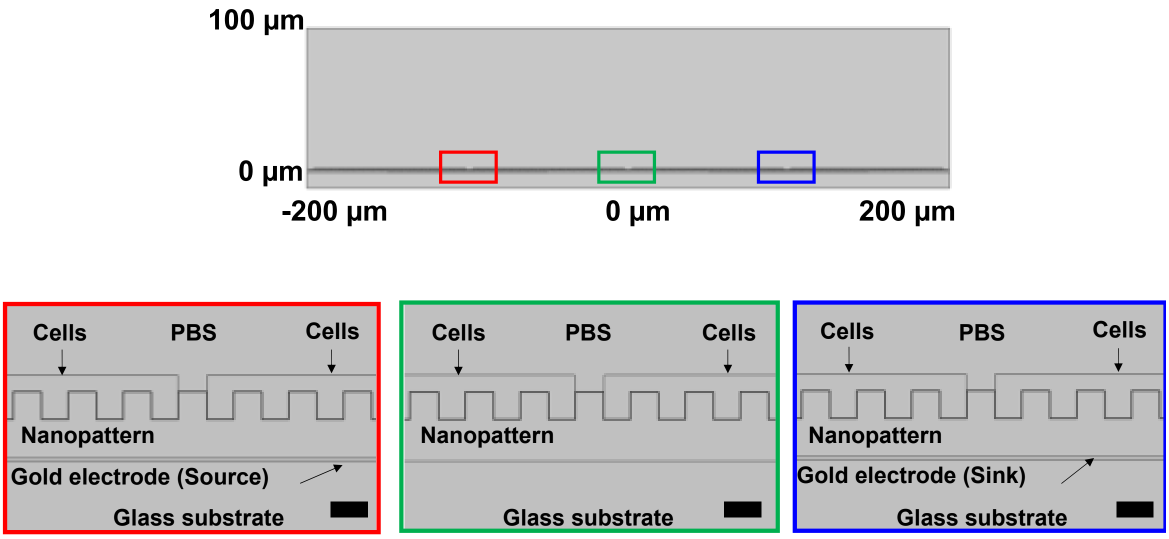

Figure S4. Numerical simulation setups for simulation analysis. Scale bars: 1 µm

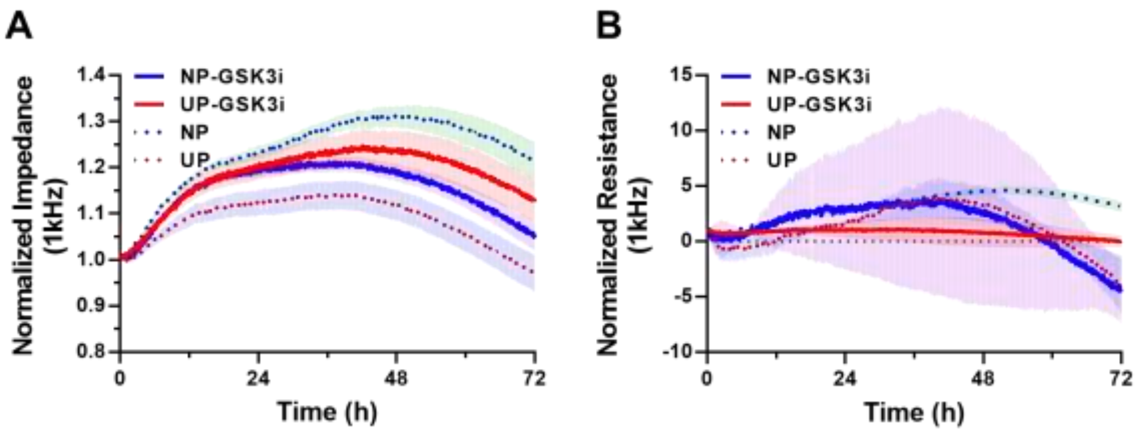

Figure S5. Real-time monitoring of (A) impedance and (B) resistance changes at low frequency (1kHz).

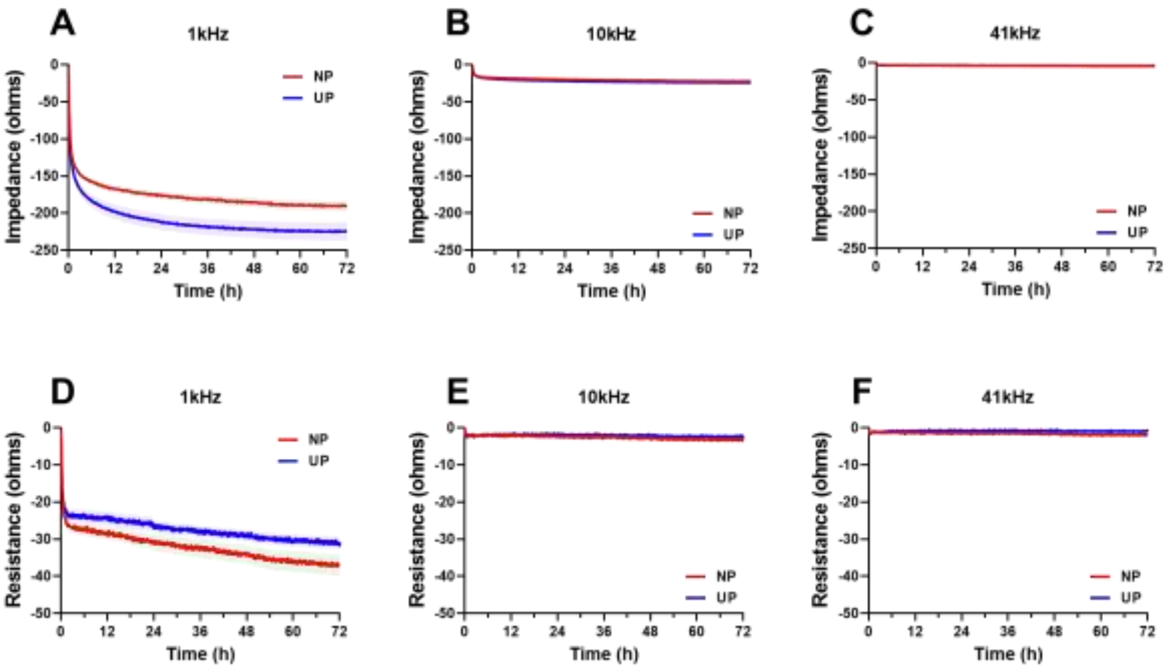

Figure S6. Impedance and resistance change when the no cell (blank controls) is applied to the IEA. (A and D) Low-frequency measurement showed negative values over time, resulting from unstable electrical movement between the electrodes. However, this can be alleviated by enhancing input frequencies..

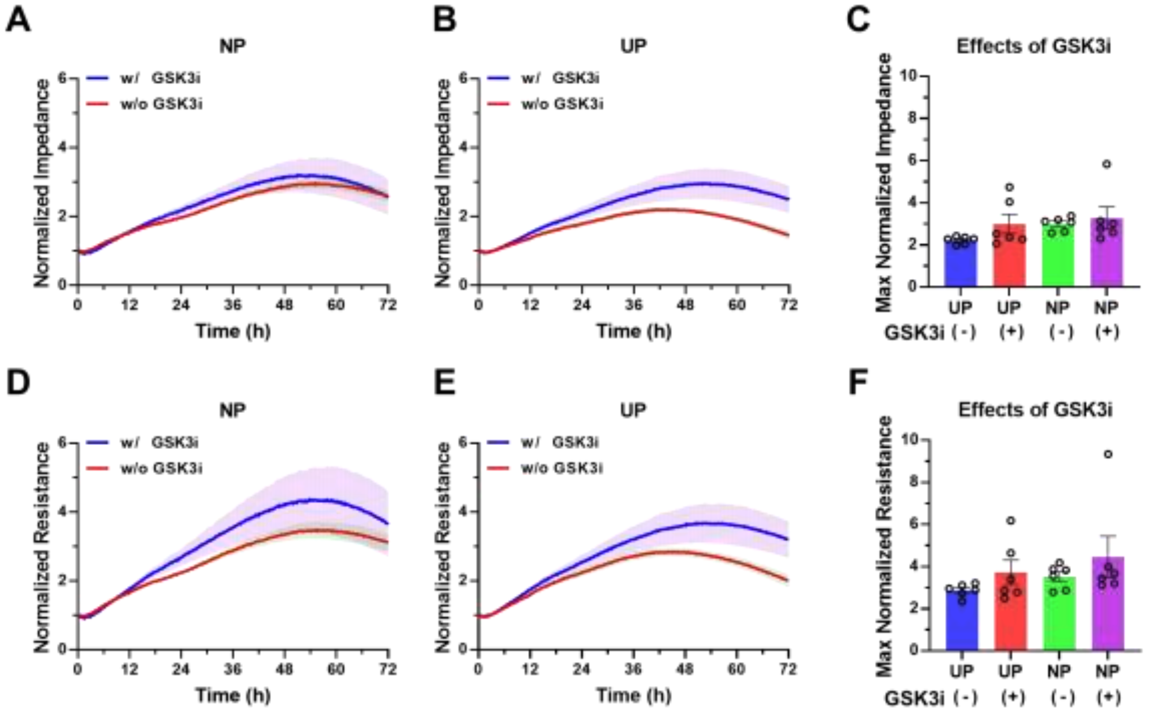

Figure S7. Real-time measurement of (A-C) impedance and (D-F) resistance changes at mid-frequency (10kHz). (C, F) Max normalized impedance and resistance.

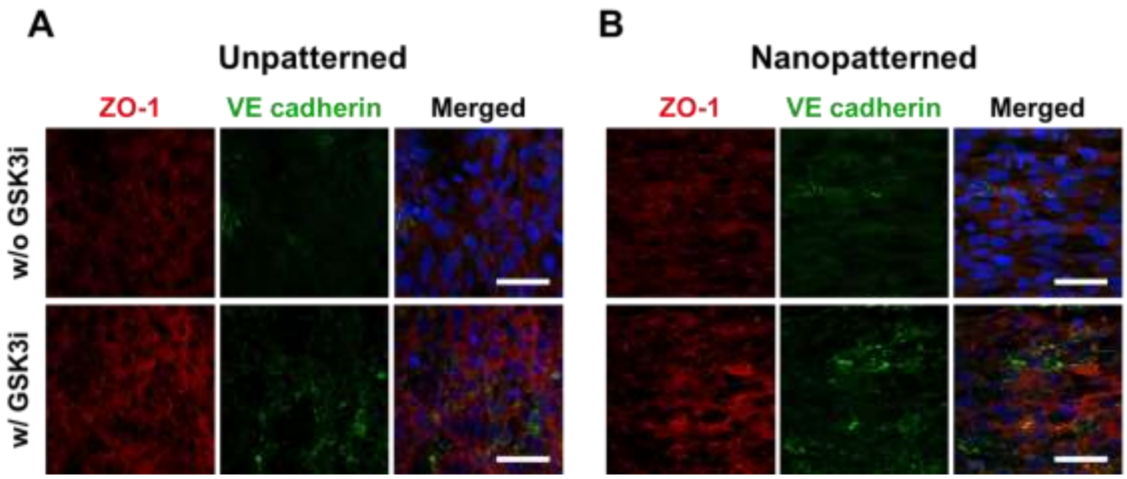

Figure S8. Effects of GSK3i on cell-cell junction protein expression in hCMECs on nanopatterned Nafion layer. (A-D) Immunocytochemistry of ZO-1, VE cadherin in hCMECs. Scale bars: 50 μm.

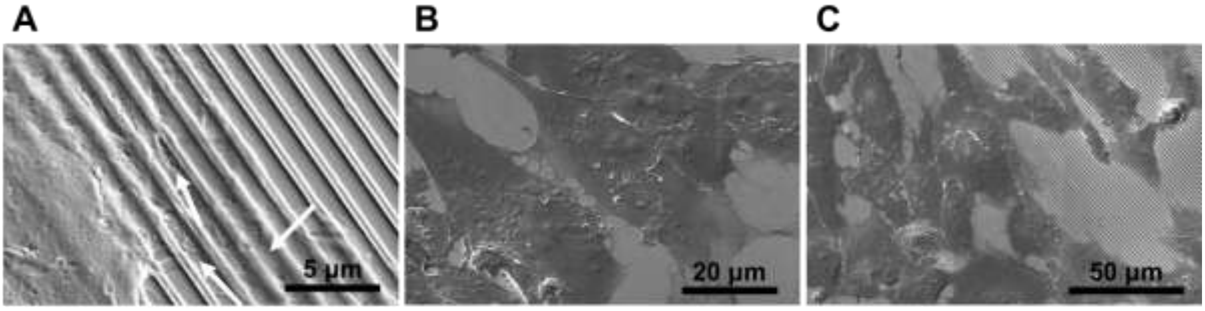

Figure S9. Endothelial cells showing (A, Arrows) filopodia and lamellipodia aligning along the nanoscale grooves. (B) Endothelial cells are cultured on a flat surface. (C) The endothelial cells are on the boundary of a pattern and flat region.

Table S1. Sequences of the primers used for RT-qPCR to measure mRNA expression.

Target Genes	Forward	Reverse
GAPDH	AGCCACATCGCTCAGACAC	GCCCAATACGACCAAATCC
CLDND1	TGAGTTTCACACTAACTGAGCAG	AGGTCCTAAGGAGATCAATCCC
TJP1	CAACATACAGTGACGCTTCACA	CACTATTGACGTTTCCCCACTC
CLDN5	CTCTGCTGGTTCGCCAACAT	CAGCTCGTACTTCTGCGACA
TITF1	AGCACACGACTCCGTTCTC	GCCCACTTTCTTGTAGCTTTCC
CDH5	AAGCGTGAGTCGCAAGAATG	TCTCCAGGTTTTCGCCAGTG
PECAM	AACAGTGTTGACATGAAGAGCC	TGTAAAACAGCACGTCATCCTT
